# Idiopathic pulmonary fibrosis: pathogenesis and management

**DOI:** 10.1186/s12931-018-0730-2

**Published:** 2018-02-22

**Authors:** Giacomo Sgalla, Bruno Iovene, Mariarosaria Calvello, Margherita Ori, Francesco Varone, Luca Richeldi

**Affiliations:** 1 Fondazione Policlinico Universitario Agostino Gemelli, Università Cattolica del Sacro Cuore, Unità Operativa Complessa di Pneumologia, Largo A. Gemelli, 8 -00168 Rome, Italy; 2 Dipartimento di Scienze Mediche e Chirurgiche, Azienda Ospedaliero-Universitaria di Modena, Università di Modena e Reggio Emilia, Struttura Complessa di Malattie dell’Apparato respiratorio , Via Del Pozzo, 71-41124 Modena, Italy

**Keywords:** Idiopathic pulmonary fibrosis, Interstitial lung disease, Diagnosis, Management, Pathogenesis, Treatment, Nintedanib, Pirfenidone

## Abstract

**Background:**

Idiopathic pulmonary fibrosis (IPF) is a chronic, progressive disease characterized by the aberrant accumulation of fibrotic tissue in the lungs parenchyma, associated with significant morbidity and poor prognosis. This review will present the substantial advances achieved in the understanding of IPF pathogenesis and in the therapeutic options that can be offered to patients, and will address the issues regarding diagnosis and management that are still open.

**Main body:**

Over the last two decades much has been clarified about the pathogenic pathways underlying the development and progression of the lung scarring in IPF. Sustained alveolar epithelial micro-injury and activation has been recognised as the trigger of several biological events of disordered repair occurring in genetically susceptible ageing individuals. Despite multidisciplinary team discussion has demonstrated to increase diagnostic accuracy, patients can still remain unclassified when the current diagnostic criteria are strictly applied, requiring the identification of a Usual Interstitial Pattern either on high-resolution computed tomography scan or lung biopsy.

Outstanding achievements have been made in the management of these patients, as nintedanib and pirfenidone consistently proved to reduce the rate of progression of the fibrotic process. However, many uncertainties still lie in the correct use of these drugs, ranging from the initial choice of the drug, the appropriate timing for treatment and the benefit-risk ratio of a combined treatment regimen. Several novel compounds are being developed in the perspective of a more targeted therapeutic approach; in the meantime, the supportive care of these patients and their carers should be appropriately prioritized, and greater efforts should be made toward the prompt identification and management of relevant comorbidities.

**Conclusions:**

Building on the advances in the understanding of IPF pathobiology, the further investigation of the role of gene variants, epigenetic alterations and other molecular biomarkers reflecting disease activity and behaviour will hopefully enable earlier and more confident diagnosis, improve disease phenotyping and support the development of novel agents for personalized treatment of IPF.

## Background

The landscape of Idiopathic Pulmonary Fibrosis (IPF), a chronic interstitial pneumonia characterized by the invariably progressive deposition of fibrotic tissue in the lungs and overall poor prognosis, has been revolutionized over the last decades by substantial advances in the understanding of disease pathobiology, the standardization of the diagnostic processes and the availability of the first treatments that modify the disease course. The increased awareness of IPF patients’ care needs, together with a thriving scenario of novel biological markers and potentially effective treatments, brought new important challenges to the surface: the need for an earlier, non-invasive and confident diagnosis, a more accurate disease stratification, and a personalized, comprehensive therapeutic approach are only some of the issues researchers and physicians will deal with over the next years. In this review, we summarize the current knowledge on pathogenesis and management of IPF, with a focus on the future perspectives in IPF care and on the challenges encountered in the translation of research outputs into clinical practice.

## Pathogenesis

**Fig. 1:**
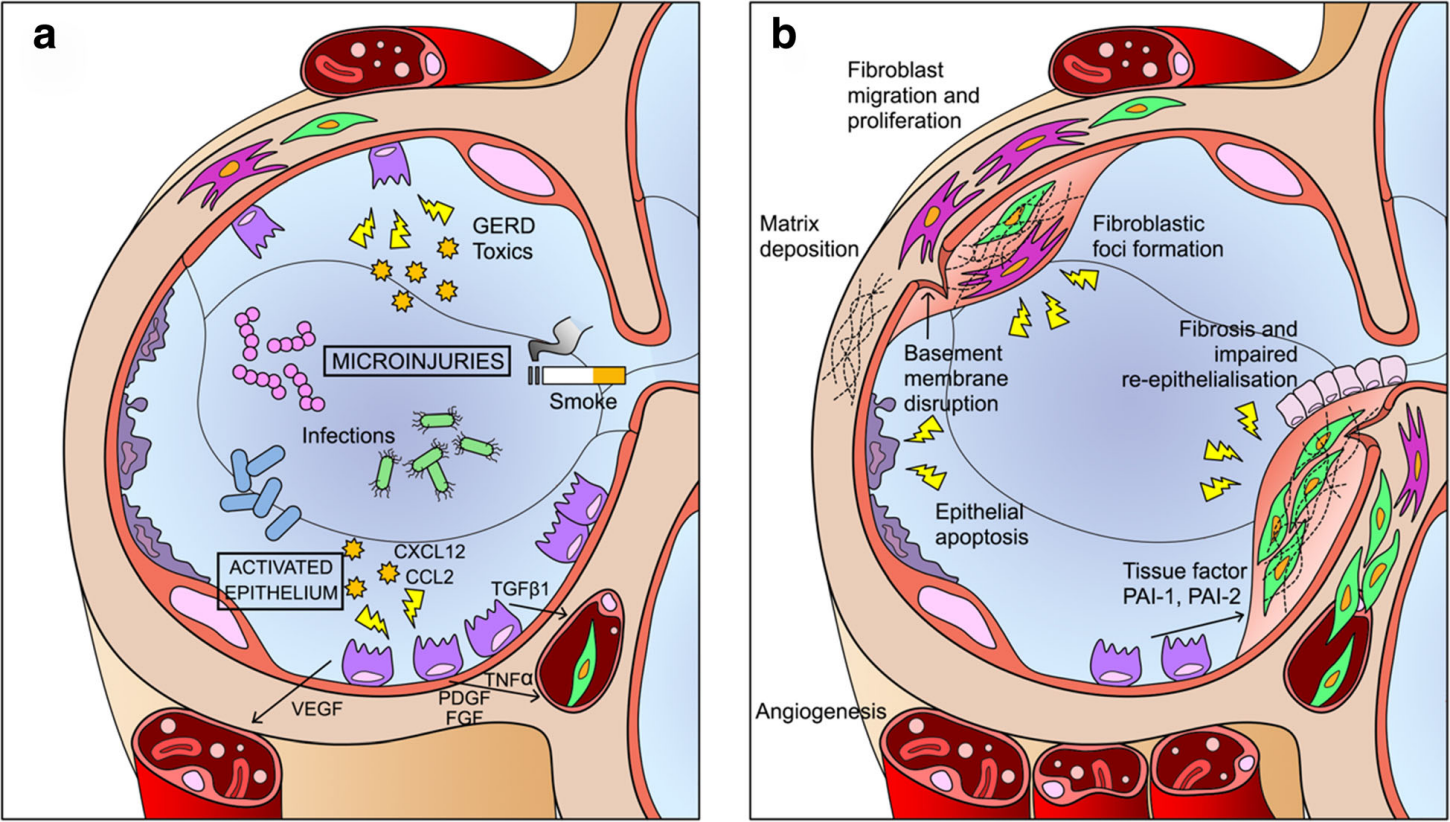
Schematic view of IPF pathogenesis. Repeated injuries over time lead to maladaptive repair process, characterized by AEC2s apoptosis, proliferation and epithelium-mesenchymal cross-talk (**a**) and following fibroblasts, myofibroblasts proliferation and accumulation of extracellular matrix (**b**).CCL2: chemokine C-C motif ligand 2; CXCL12: C-X-C motif chemokine 12; FGF: fibroblast growth factor; PAI-1: plasminogen activator inhibitor 1; PAI-2: plasminogen activator inhibitor 2; PDGF: platelet-derived growth factor; TGF-β1: Transforming Growth Factor-Beta 1; TNF-α: tumor necrosis factor-alpha; VEGF: vascular endothelial growth factor

Despite the comprehensive understanding of IPF pathogenesis remains elusive, research efforts in the last few years have reached important milestones. Several environmental and microbial exposures have been proposed as playing roles in IPF pathobiology that might be far from collateral, making the concept of “idiopathic” less compelling. Individual genetic and epigenetic factors remain the most important for the development of the fibrotic process, although the contribution of the variants so far identified, or their interaction with the putative external factors has yet to be clarified. In this context of genetic susceptibility, the repeated micro-injury of the alveolar epithelium has been recognized as the first driver of an altered repair process where several lung cells develop aberrant behaviours, leading to the development and sustainment of the fibrotic process. This section will cover in detail the current evidence on the contribution of these factors to IPF pathogenesis and the main goals of research for the years to come. The main pathogenetic actors in IPF are also illustrated in Fig. 1.

## Risk factors

### Environmental

Several epidemiological studies have demonstrated that environmental exposures are involved in the pathogenesis of IPF. Although evidences of dose-response relationship are limited, findings have strongly associated cigarette smoking and metal dust with the risk of IPF, even for the familial form of pulmonary fibrosis [1]. Even after smoking cessation, smoke remains a risk factor by inducing a self-sustaining lung injury. Moreover, IPF patients with cigarette smoking history have a poorer survival compared to non-smokers [2].

Further significant correlations have been established among IPF and agriculture and farming, livestock, wood dust and stone, sand and silica [3].

Microbial agents (viral, fungal, and bacterial) play a potential role in the pathogenesis of IPF [4]. An imbalance in bacterial community composition has been observed in patients with interstitial lung disease, when compared with healthy lungs. Studies suggested that the analysis of IPF lung microbiome composition may provide an explanation for disease pathogenesis and may be useful as a prognostic biomarker [5]. Intriguingly, patients expressing a Mucin 5B(MUC5B) minor allele genotype had significantly lower bacterial load compared with IPF patients without this genotype [6].

Moreover, Huang and co-workers analysed the patients enrolled in the COMET-IPF study and demonstrated a relationship between peripheral blood immune gene expression and bronchoalveolar lavage (BAL) microbiome features in IPF [7]. Viral infections, such as Epstein-Bar-virus, cytomegalovirus, hepatitis C virus, and human herpesvirus-8, were frequently found in the lungs of IPF patients and therefore considered to be risk factors. Nevertheless, evidences on the contribution of these virus showed conflicting results [8].

Studies investigating drugs as antivirals, antibiotics, and antifungals have shown a great promise for IPF treatment, consolidating the link between microbiome and IPF [9].

### Genetic

Susceptibility to IPF is probably related to several genetic features characterized by a combination of gene variants and transcriptional changes, that result in the loss of epithelial integrity. Familial interstitial pneumonia (FIP) is identified when two or more member of the same biological family are affected [10]. FIP is inherited in an autosomal dominant trait with variable penetrance and accounts from 2% to 20% of the overall cases of idiopathic interstitial pneumonias [11]. Rare genetic variants have been reported by different studies performed on large population of FIP [12, 13]. These variants, implicated in maintenance of telomere length (Telomerase reverse transcriptase-TERT, Telomerase RNA component-TERC, Poly(A)-specific ribonuclease-PARN, and regulator of telomere elongation helicase-RTEL)and surfactant dysfunction (Surfactant Protein C and A2-SFTPC, SFTPA2), have been recognized even among those with sporadic disease [14]. Moreover, two large genome-wide association studies (GWAS) have identified common genetic variants, crucial for the epithelial integrity, as risk factors of IPF [15, 16]. These studies identified the potential importance of telomere biology (TERT, TERC, OBFC1), host defence (MUC5B, ATPase phospholipid transporting 11A–ATP11A, toll interacting protein-TOLLIP), and cellular barrier function (desmoplakin-DSP, dipeptidyl peptidase 9-DPP9) for the development of the disease. Both the GWAS established the role of the promoter of MUC5B gene as a risk factor of disease and characterized other common variants associated with IPF, e.g. TOLLIP and Toll-like receptor (TLR) 3. Nevertheless, the MUC5B promoter region rs35705950, a common gain-of-function variant with low penetrance, has been confirmed as the strongest risk factor for development of both familial interstitial pneumonia and sporadic IPF [17–19]. Subjects affected by IPF with the variant rs35705950 have shown a better survival compared with patients without this variant [20].

### Epigenetic alterations

Any process that modifies gene activity without changing the underline genetic code is defined as epigenetic alteration [21]. Traditionally, epigenetic modifications refer to DNA methylation and histone modifications. Besides, non-coding RNAs (especially microRNAs) dysregulation has been recently included as part of epigenome. The leading mechanisms of DNA methylation and histone modifications seem to mediate both genetic and environmental influence on gene expression and disease features, especially with age. Increasing evidences support a central role for epigenetic alterations in IPF [22, 23]. DNA methylation changes consist of both hyper- and hypo-methylation of cytosine residues in different genes, with accidental errors in methylation [24]. A genome-wide DNA methylation analysis of lung tissue involving 94 patients with IPF and 67 controls, recognized 2130 genome-wide differentially methylated regions, of which about a third were associated with significant changes in gene expression, including IPF-associated common genetic variants [25]. MicroRNAs silence almost 90% of human genes through degradation of target mRNA or inhibition of protein translation. Evidences have identified significant changes in the levels of regulatory miRNAs in IPF patients when compared with healthy subjects [26].

Cigarette smoking and ageing are the main effectors of epigenetic modifications, given their association with IPF and the relationship between them and DNA methylation [24, 27]. Stochastic changes in DNA methylation produce epigenetic mosaicism in ageing stem cells as shown by genome-wide studies in aging cell and tissue. This epigenetic drift could theoretically limit cell plasticity leading to the development of age-related diseases such as IPF [24, 27].

### Ageing

Ageing is a physiological progression to the death, through loss of function and increasing weakness. Cellular and clinical age-related changes play a leading role in IPF [28]. Age-related cellular changes primarily affect the alveolar epithelium. Nine hallmarks contributing to the aging process have been suggested: genomic instability, loss of telomere protective functions, epigenetic changes, loss of proteostasis, deregulated nutrient sensing, mitochondrial dysfunction, cellular senescence, stem cell exhaustion, and altered intercellular communication. Epithelial cell senescence induces pulmonary fibrosis through both the abnormal secretory pattern of the lung epithelium and the increased resistance to apoptosis of myofibroblasts [29]. Naturally aged experimental models (wild-type mice) exhibit a more severe fibrotic response to environmental stimuli and injury, compared to younger mice [30]. A recent paper suggests that fibroblasts from lungs of old mice express a fibrogenic phenotype that leads to resistance to apoptosis and increased susceptibility to fibrotic response after injury. These findings have been partially associated with an increased expression of plasminogen activator inhibitor 1 (PAI-1), which is an effector of Transforming Growth Factor-Beta 1 (TGF-β1), a key factor in the development of senescence through the induction of p21 [31].

## Cells and mediators

In the last years research efforts have been oriented to the pathobiology of IPF. Previously defined an inflammatory disease, IPF is currently considered an epithelium-driven disease, in which a dysfunctional, ageing lung epithelium exposed to recurrent microinjuries lead to defective attempts of regeneration and aberrant epithelial-mesenchymal crosstalk, creating an imbalance between profibrotic and antifibrotic mediators, maintaining an environment supportive of exaggerated fibroblast and myofibroblast activity and hijacking the normal reparative mechanisms to chronic fibroproliferation. In this section we will focus on the different cell type and mediators involved in IPF pathogenesis [32].

### The concepts of dysfunctional epithelium and aberrant wound healing process

It is postulated that fibrosis evolves over a long interval of time in patients with IPF; when diagnosed, lung structure is importantly modified by disease, and pathological features are characterized by different stages of epithelial damage, AEC2s hyperplasia, dense fibrosis, abnormal proliferating mesenchymal cells. What happens before diagnosis is still partly unclear, but the current theory is that a dysfunctional ageing epithelium is the key to understand IPF pathogenesis [33].

In normal lungs, loss of AEC1s after an injury is followed by proliferation and differentiation of AEC2s and stem cells, that restore alveolar integrity involving several mechanisms: coagulation cascade, new vessels formation, fibroblast activation and migration, collagen synthesis and proper alignment. Many chemokines, as TGF-β1, platelet-derived growth factor (PDGF), vascular endothelial growth factor (VEGF) and fibroblast growth factor (FGF), lead the process. If injury persists, or the ability to restore normality is impaired, the wound healing process will pass through an inflammatory phase, with increased levels of interleukin-1 (IL-1) and tumor necrosis factor-alpha (TNF-α), creating a biochemical environment leading to chronic abortive regeneration and tissue remodelling [34].

In IPF patients lung epithelium is thought to be dysfunctional, and genetically susceptible to aberrant response to injuries. Possible genetic causes of dysfunction have been discussed in the precedent section. These mutations affect genes expressed in AECs. Altered genetic expression, with consequent aberrant transcription and translation, leads to abnormal protein production, potentially able to damage cellular environment and alterate cellular behaviour, and to accelerated cellular senescence. The result of these abnormalities is a fragile epithelium, with reduced ability to respond to an injury [32, 35, 36].

### AEC2s and the initiation of maladaptive repair process

The repetitive exposure of alveolar epithelium to microinjuries, as infections, cigarette smoke, environmental inhaled toxics, gastro-oesophageal reflux lead to damage of AEC1s. Dysfunctional AEC2s should regenerate damage cells, but their ability to re-establish normality is seriously impaired; this is the crucial moment of IPF pathogenesis [37, 38].

The cellular activity leads to protein over-expression and endoplasmic reticulum stress (ERS), a protective pathway that occurs when there is an imbalance between cellular demand for protein synthesis and the endoplasmic reticulum capacity to work properly. The consequence is the activation of another protective pathway, the unfolded protein response (UPR), designed to re-establish normality in endoplasmic reticulum(ER) work inhibiting proteins translation and targeting them for degradation, but also to lead cell to apoptosis if stress persists. The activation of UPR has several consequences on cellular behaviour, not completely understood. A relevant consequence is the activation of intracellular apoptotic pathways; furthermore, UPR stimulates the production of profibrotic mediators, as TGF-β1, PDGF, CXCL12 (C-X-C motif chemokine 12), CCL2 (chemokine C-C motif ligand 2) [36, 39].

TGF-β1 is probably the most important mediator involved in IPF pathogenesis. AEC2s may produce it as a consequence of actin/myosin-mediated cytoskeletal contraction induced by UPR, through ανβ6 integrin activation. Theανβ6 integrin/TGF-β1 pathway is a fundamental biological process: the molecules are constitutively bound, suggesting that the system is primed to detect injurious stimuli. TGF-β1 may be activated even by lysophosphatidic acid (LPA), whose production is controlled by autotaxin. TGFβ1 is a strong pro-fibrotic mediator: it promotes epithelial cell apoptosis, epithelial mesenchymal transition (EMT), epithelial cell migration, production of other profibrotic mediators, circulating fibrocytes recruitment and fibroblasts activation, proliferation and transformation into myofibroblasts, production of VEGF, CTGF (connective-tissue growth factor) and other pro-angiogenic mediators and several other pathways [40].

The EMT is a molecular reprogramming of AEC2s, induced by UPR and enhanced by pro-fibrotic mediators and pathways. Epithelial cells express mesenchymal cell-associated genes, become detached from basement membrane, migrate and down-regulate their typical markers. The most characteristic marker of these transitional cells is αSMA (alpha smooth-muscle actin), typical of myofibroblasts. Such an event may occur in three different moment of cellular and tissue life: development, cancer and fibrosis, whereas it is not required to restore normality during wound healing response [36].

Other key pathways in IPF are a group of deregulated embryological programs, as the Wnt-β-catenin signalling, involved in EMT and fibrogenesis and activated by TGF-β1, Sonic Hedgehog (Shh), gremlin-1, and phosphatase and tensin homologue (PTEN). Deregulation of these pathways confers resistance to apoptosis and proliferative advantages to cells [35].

The UPR, TGF-β, and EMT are activated in patients with IPF, but how this activation occurs is still widely undefined. Many possible causes of ERS and UPR have been identified, as Herpes virus infection end epigenetic effects of inhaled toxic [39]. An intriguing hypothesis to explain why cells undergo ERS and UPR is based on genetic abnormalities leading to over-expression of proteins or to production of misfolded ones, as the gain-of-function promoter variant in MUC5B rs35705950 or mutation of surfactant protein encoding gene. The considerable amount of proteins produced by AECs and basal stem cells during the regeneration of damaged epithelium may lead to ERS; in such a delicate moment of cellular lifeERS and consequent UPR may disrupt developmental pathways and hijack the normal reparative mechanisms to chronic fibroproliferation. Other possible roles of excessive mucin production could involve impaired mucociliary function or mucus composition, as shown by *Evans* and co-workers [38, 41].

### The role of the endothelium and coagulation cascade

The damage to alveolar structure and the loss of AECs, with disruption of basement membrane, involves alveolar vessels and leads to increased vascular permeability. This early phase of wound healing response is characterized by the formation of wound clot; consequently, new vessels should be formed and endothelial cells should proliferate, involving endothelial progenitor cells (EPCs). *Malli* and co-workers demonstrated that IPF patients present significantly decreased EPCs, with important consequences as failure of re-endothelization, that may lead to a dysfunctional alveolar-capillary barrier, inducing a pro-fibrotic response, and compensative augmented levels of VEGF, that might consequently stimulate fibrotic process and abnormalities of vessels function, contributing to cardio-respiratory physiologic consequences typical of advanced stage of disease. Furthermore, endothelial cells may undergo a mesenchymal transition, with the same consequences of EMT [42].

Endothelial and epithelial damage lead to activation of the coagulation cascade in the early phases of wound healing process. Coagulation proteinases have several effects on cells involved in wound healing. The tissue factor (TF)-dependent pathway is the most important in IPF pathogenesis, leading to a pro-coagulation state enhanced by increased levels of plasminogen activation-inhibitors as PAI1 and PAI2, active fibrinolysis inhibitors and protein C-inhibitors. The pro-coagulation environment reduces the degradation of extracellular matrix(ECM), resulting in a profibrotic effect, and inducing differentiation of fibroblasts into myofibroblasts via proteinase-activated receptors [34, 35].

### The bronchiolisation of alveolar spaces

AECs are not the unique cells whose behaviour is modified during IPF pathogenesis: also airway basal cells change their biological program to respond to persistent injuries and epithelial damage. This regenerative response activates developmental pathways and leads to an aberrant proliferation with irreversible changes in alveolar spaces architecture. This phenomenon, known as bronchiolisation of alveolar spaces, has important functional consequences and is one of the pathological features of usual interstitial pneumonia (UIP) [32].

### Mesenchymal cells and extracellular matrix

The contribution of mesenchymal cells, and particularly of fibroblasts and myofibroblasts is crucial for IPF pathogenesis; these cells are recruited, activated and induced to differentiate, trans-differentiate and proliferate by the abnormal biochemical environment created by activated epithelial and endothelial cells. It is still unknown the initial trigger and source of mesenchymal cell recruitment, but current literature agrees to define fibroblasts and myofibroblasts as the key player cells of IPF pathogenesis. Mesenchymal cell-type involved are circulating fibrocytes, pulmonary fibroblasts and myofibroblasts [43].

### Fibrocytes

Fibrocytes are circulating bone-marrow derived mesenchymal cell progenitors, co-expressing CD45 or CD34 withtype-1 collagen. They could be recruited by damaged tissues when pathological processes significantly deplete local mesenchymal cells. The activated epithelium recruits them exposing CXCL12, CCL2 and secreting TGF-β1, a strong fibrocytes activator that induces αSMA production. In damaged lung, fibrocytes contributes to IPF through ECM production differentiating into fibroblasts and myofibroblasts and enhancing profibrotic environment by secreting profibrotic cytokines. Furthermore, there are increasing evidences that percentage of circulating fibrocytes increases during acute exacerbation of IPF (AEIPF), and afterwards decreases when the hyper-acute phase ends. Therefore, they could be considered promising biomarkers, with prognostic implications [44].

### Fibroblasts, myofibroblasts and extracellular matrix

Fibroblasts are tissue mesenchymal cells committed to re-establish a normal and well-structured ECM in wound healing repair process. During IPF pathogenesis both lung and fibrocytes-derived fibroblasts are persistently exposed to profibrotic mediators secreted by activated fibroblasts, leading to ECM production and trans-differentiation to myofibroblasts. The most important stimulating factor for trans-differentiation is TGF-β1, but also PDGF plays a significant role. The activation of these cells has important consequences on their intra and extracellular behaviour: IPF lung fibroblasts share unique properties, as a hyper-methylated DNA profile, that enhance genetic transcriptions; this behaviour has similarity to lung cancer biology [39].

It is still debated if deregulated AECs undergoing EMT and expressing αSMA could be considered myofibroblasts or not. Myofibroblasts synthesize more ECM than fibroblasts. The matrix produced by myofibroblasts is poorly organized but very dense. Moreover, they persist longer than fibroblasts in damaged tissue. Myofibroblasts have contractile properties due to αSMA, similarly to smooth muscle cells (SMCs); the main difference between these cells is the irreversibility of contraction of myofibroblasts, that may regulate collagen remodelling, inducing a spatial re-organization of collagen fibrils, increasing mechanical stress and leading to a stiffer ECM. The mechanical characteristics of deposed ECM is probably the most important factor in regulation of myofibroblasts activity: in fact, their synthesis activity is enhanced by contact with a stiffer matrix, creating a positive feedback loop, whereas a healthy soft substrate strongly inhibits myofibroblasts and leads to reduction of their number. This is probably the reason of absence of myofibroblasts in normal tissues [43, 45, 46].

### Fibrobastic foci and epithelium-mesenchyma crosstalk

A typical pathological feature of UIP is the presence of fibroblastic foci (FF), small clusters of active fibroblasts and myofibroblasts, very close to hyperplastic AEC2s. The strict association between AEC2s and mesenchymal cells inside the FF favours aberrant crosstalk, and enhances effects of TGF-β1, PDGF, Wnt pathway, amplifying the profibrotic environment and leading to a greater trans-differentiation rate, acquisition of invasive characteristics and exaggerated matrix production, with an imbalance between deposition and degradation of collagen.

### Inflammation and immunity

The pathobiology of IPF is leaded by aberrant epithelial-mesenchymal crosstalk, but the inflammation may play an important role. Inflammatory cells are involved in normal wound healing since early phases. Macrophages immediately produce cytokines that stimulate an inflammatory response, and later participate to the transition to a reparative environment, recruiting fibroblasts, epithelial and endothelial cells. When injury persists, neutrophils and monocytes are recruited. The production of reactive oxygen species (ROS) worsens epithelial damages, and an imbalance between antioxidants and pro-oxidants may lead to epithelial cells apoptosis and dysfunctional pathways activation. Monocytes and macrophages produce PDGF, a strong profibrotic mediator; furthermore, CCL2 and the macrophage colony stimulating factor (M-CSF), also known as colony stimulating factor 1 (CSF1) may have direct profibrotic effects [37, 39].

The role of lymphocytes is still debated; certain lymphocytic cytokines are considered profibrotic, with direct effects on fibroblasts and myofibroblasts activity. Th-1, Th-2 and Th-17 T-cells have been linked to IPF pathogenesis. Th1 subset produces IL-1α, TNF-α, PDGF andTGF-β1, with a final profibrotic effect, butTh2 and Th17 responses seems to be more important in IPF pathogenesis. Th2 subset typical interleukin is IL-4. This interleukin induces increased levels of IL-5, IL-13 and TGF-β1, recruiting macrophages, mast-cells, eosinophils and mesenchymal cells, and is directly implied in fibroblasts activation. Furthermore, fibroblasts isolated from patients with IPF show hyper-responsiveness to IL-13; this interleukin has a positive effect on fibroblasts’ activity, enhancing ECM production. Th17 subset indirectly promotes fibrosis, increasing TGF-β1 levels; it is positively regulated by TGF-β1, creating a positive feedback loop [34]. In both bronchoalveolar lavage fluid and peripheral blood of IPF patients there is a decreased number of CD4+ CD25+ FOXP3+ regulatory T cells (Tregs) compared with healthy people and patients with other lung diseases. Tregs play a crucial role in immunologic tolerance and prevention of autoimmunity; their numerical and functional deficiency may play a central role in the initial phases of IPF pathogenesis, as shown by *Kotsianidis* and co-workers [47].

In IPF lung levels of Interferon-γ (IFN-γ) are low. IFN-γ inhibits fibroblastic activity and switch off Th2 response. However, novel studies on the role of inflammation in IPF pathobiology are needed, and their results will probably either help to understand what happen in the early stages or contribute to clarify the mechanisms of disease progression [39].

## Clinical management

### Diagnosis

#### Current diagnostic approach

**Fig. 2:**
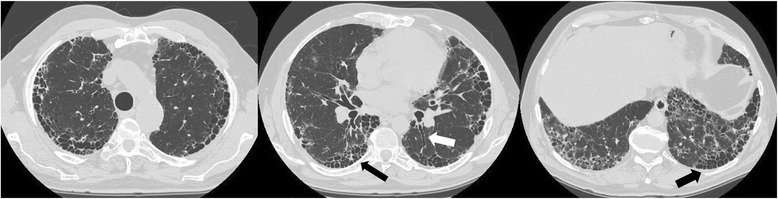
Typical Usual Interstitial Pneumonia pattern on high resolution computed scan sections showing upper, middle and lower lung regions from left to right. Black arrows indicate subpleural honeycombing; white arrow indicates traction bronchiectasis

According to the 2011 joint statement by the American Thoracic Society (ATS), European Respiratory Society (ERS), Latin America Thoracic Association (ALAT) and Japanese Respiratory Society (JRS) [48], the diagnosis of IPF can be secured by the presence of a UIP pattern on High Resolution Computed Tomography (HRCT) (Fig. 2) or by specific combinations of radiologic and histopathologic patterns in patients undergoing surgical lung biopsy. Any plausible cause of secondary interstitial involvement should be carefully excluded by means of a thorough medical history and other procedures such as laboratory tests or BAL when necessary. Multidisciplinary discussion (MDD) among different experts (including clinicians, radiologists, pathologists, and rheumatologists and thoracic surgeons in selected cases) has demonstrated to improve interobserver agreement and diagnostic accuracy and is therefore recommended before the final diagnosis is made [48]. The 2011 document provided for the first time a rigorous, standardized, evidence-based diagnostic framework useful for clinicians and researchers [49]. After the positive end-points of nintedanib and pirfenidone [50, 51] in the clinical trials, the treatment guideline was updated in 2015 [52]. Nevertheless, the applicability of current diagnostic criteria in daily clinical practice proved to be challenging over the last years.

Firstly, around 10% patients presenting with an atypical UIP pattern on HRCT cannot undergo surgical lung biopsy due to age, advanced disease, or poor clinical conditions [53]. These patients do not fall into any diagnostic category and are kept from getting access to the available treatments. Alternative, allegedly less-invasive procedures such as transbronchial cryobiopsy have been proposed to provide high diagnostic confidence when performed by experienced operators [54, 55]. The radiologic UIP pattern as defined by the current guidelines represents itself a source of uncertainties. The evaluation of honeycombing, required for the diagnosis of UIP on HRCT, is prone to significant interobserver variability even among interstitial lung disease (ILD)-expert radiologists [56]. The interobserver agreement for a radiologic UIP-pattern diagnosis among thoracic radiologists has been proven to be only moderate, irrespective of the level of experience [57]. Retrospective analyses from IPF trials showed that possible UIP on HRCT is frequently associated with a histopathologic pattern of definite or probable UIP, and even a HRCT pattern inconsistent with UIP could be in many cases associated with a definite or probable UIP pathological diagnosis [58, 59]. Recently, a post-hoc subgroup analysis of patients from the nintedanib phase 3 program INPULSIS has shown that patients with a possible UIP pattern on HRCT and no surgical lung biopsy confirmation do progress similarly to patients with a definite radiologic UIP and/or confirmation by surgical lung biopsy, and that both subgroups respond in a similar way to treatment with nintedanib [60]. The 2011 statement also neglects the potential diagnostic role of clinical and demographic features (e.g., age, familiarity, disease behaviour), which have not been incorporated in the diagnostic algorithm [61]. Indeed, such approach is not consistent with how clinical thinking operates, andaconsistent body of evidence suggests that the simple combination of readily available clinical data such as older age (e.g. > 70 years) with different patterns of interstitial involvement on HRCT is highly predictive toward the confirmation of UIP at subsequent histology [28, 62].Recently, a Fleischner Society working group has published an update of the diagnostic approach of IPF. Major changes have been proposed in the diagnostic HRCT categories, with the aim of aiding clinicians to providepatients with a more confident diagnosis, even without tissue confirmation. In this new context, the lack of honeycombingbut the presence of reticular pattern with peripheraltraction bronchiectasis is defined as “probable UIP”, and in the right clinical scenario does not require a SLB for confirmation of UIP.Biopsy is thus reserved to patients with evidence of fibrosis that lack specificity in distribution and pattern (indeterminate for UIP), and to patients with CT picture most consistent with a different diagnosis. Another key feature of the guidelines is to incorporate disease behaviour in the diagnostic work-up. Hence, a working diagnosis of IPF should be confidently formulated by MDD in selected cases. Most important, all diagnoses should be reviewed over time to increase confidence [63].

Finally, despite the universally recognized utility of MDD, there is no reference standard to measure its validity, and a consensus on what constitutes reasonable purposes, structure, and governance of MDD meetings has been recently advocated [64]. Moreover, the vast majority of MDD evaluations concerns diagnosis, whereas decisions regarding disease management are still often taken by the pulmonologist alone [48, 64].

#### Future perspectives

The recent advances in the treatment of IPF make the need to ameliorate the diagnostic process more compelling than ever, with the goal of limiting the number of unclassifiable ILD and enhancing the identification of patients who can benefit from treatment. Groups of experts have recently indicated the need for a more comprehensive and dynamic diagnostic process, integrating the contributions of all available clinical data and diagnostic techniques. Physicians should be allowed establish diagnoses when diagnostic confidence is sufficiently high based on available criteria and clinical judgment, while provisional diagnoses should be made when there is a leading diagnosis which does not strictly meet all the criteria. In order to address the issue of unclassifiable cases, complementary information to traditional histopathology might be provided by modern technologies such as micro-computed tomography (micro-CT or μCT), a novel imaging technique that allows the three-dimensional study of paraffin-embedded tissue blocks [65]. Preliminary evidence suggests that this approach could provide additional information as compared with traditional two-dimensional histopathology as to the distribution and morphology of key diagnostic features such as fibroblastic foci, thus potentially increasing diagnostic accuracy [66]. Finally, the future ideal algorithm should be open to the inclusion of novel techniques investigating the molecular signature of the disease [61, 67]. The use of genetic and biological markers to assist the diagnostic process in IPF is indeed an appealing approach, and would allow stratify patients based on clinical behaviour and response to drugs, thus optimizing disease management and enhancing the efficacy of clinical trials [68]. A novel, intriguing possibility for improving diagnostic accuracy for UIP with less invasive methods is the genomic analysis and machine learning approach applied on transbronchial biopsies [69]. Several genetic variants, either rare and common, have been associated with IPF by genome-wide association studies [15, 70–73]. While the biological effect of the genetic variants isolated so far and the influence of environmental factors toward the development of a progressive fibrosis remain largely unknown [32], the results of some large population sequencing studies are awaited soon and will hopefully allow a deeper understanding of the penetrance and effect sizes of common and rare genetic variants toward distinct clinical phenotypes. One example is the PROFILE (Prospective Observation of Fibrosis in the Lung Clinical Endpoints) study, a large prospective cohort study designed to identify IPF phenotypes and endotypes and find out new diagnostic and prognostic instruments based on a precision medicine approach [74]. Recently an analysis from the PROFILE study identified epithelial-secreted serum proteins that could be used to predict disease progression [75]. It is conceivable that in the near future all these mechanistic data, coming from biosamples, should be incorporated in the context of a multidisciplinary diagnostic process, alongside clinical and morphologic impressions. A list of possible biomarkers is available in Table 1.

**Table 1:** Most relevant biomarkers *(Authors’ note: the table should be placed between the paragraphs “future perspectives” and “treatment”)*

Group	Subgroup	Markers	Description
Dysfunctional alveolar epithelial repair/cellular senescence abnormalities	Surfactant proteins	SP-A SP-D	Apolipoproteins produced by alveolar type 2 cells. Mutations within genes that codify these proteins, determining increasing of their levels, are associated with worst prognosis
	Mucin family	Mucin 5B	Mucin 5B is a cytoplasmic protein encoded by the MUC5B gene. This protein is highly expressed in distal airways, respiratory bronchioles and honeycombing cysts of patients with IPF; furthermore, a single nucleotide polymorphism (SNP) in the promoter region of the gene (rs35705950) is a strong risk factor for developing IPF
	Telomerase complex	Telomerase reverse transcriptase (TERT) Telomerase RNA complex (TERC)	In IPF and familial pulmonary fibrosis there is a reduction of telomeres length both in lung tissue and in peripheral blood. Mutations in TERT and TERC genes play an important role, and are associated with about 1–3% of sporadic IPF and 7–15% of familial interstitial pneumonia. In families with IPF, several telomerase mutations may be found in 15–20% of cases. These mutations are associated with reduced survival
	MicroRNAs (miRNAs)	Many different types	miRNAs are short endogenous non-coding RNA molecules, which may influence cellular differentiation, morphogenesis or apoptosis, modifying cellular activity. Fibroblasts and alveolar epithelial cells may undergo significant changes in their function, as epithelial-mesenchymal transition and senescence, interacting with miRNAs
	Integrin family	αvβ6 αvβ3 αvβ5 αvβ1	Trans-membrane receptors involved in relationship between cellular membrane and cytoskeleton with ECM. They may activate TGFβ and induce collagen production. αvβ6 integrin is over-expressed in IPF patients, and may be use as diagnostic and prognostic markers. Furthermore, it is a potential therapeutic target
	Reactive oxygen species (ROS)	Anion super-oxide (O_2_^−^) Hydrogen peroxide (H_2_O_2_)	An excessive and prolonged exposure of cells to oxidative stress may lead to fibrosis promoting endoplasmic reticulum stress and apoptosis. Patients with IPF probably have decreased levels of antioxidant defences, as catalase, glutathione and superoxide dismutase
ECM remodelling	Matrix metalloproteinases (MMPs)	MMPs 1–2–3-7- 8-9	MMPs are endoproteases that participate to ECM homeostasis. Microarray techniques in peripheral blood and bronchoalveolar lavage fluid of IPF patients may show high expression of these biomarkers; intriguingly, IPF patients have increased values of MMP-1 and 7 compared to other ILD-patients, suggesting the possibility to use a relatively simple analysis in differential diagnosis
	Lysyl oxidases (LOXs)	LOXL2	LOXs are enzymes involved in homeostasis of type I collagen; their activity results in a major stiffness of fibrillar collagens, increasing local matrix structural tension and activating fibroblast and TGFβ_1_ signalling.
		Periostin	Periostin is a fibroblast-secreted ECM protein, involved in adhesion and migration of epithelial cells. In IPF patients it correlates with functional decline.
Fibroblast activation/proliferation	Fibrocytes		Fibrocytes (CD45 and CD34-positive) differentiate into fibroblasts and myofibroblasts if attracted to injured tissues by chemokines and growth factor. Patients with IPF have increased level of circulating fibrocytes. A fibrocyte chemokine receptor (CXCL12) is increased in peripheral blood of IPF patients, correlating with lung function.
		Connective tissue growth factor (CTGF)	CTGF is involved in connection between cell membranes and ECM, cell proliferation, angiogenesis and ECM production.
		Galectin-3	Molecule involved in fibroblast proliferation, activation and in collagen synthesis, exacerbating ECM deposition and fibrosis.
		Fibulin-1	Molecule involved in fibroblast proliferation, activation and in collagen synthesis, exacerbating ECM deposition and fibrosis.
		Osteopontin	Molecule involved in fibroblast proliferation, activation and in collagen synthesis, exacerbating ECM deposition and fibrosis.
Immune dysregulation/inflammation		Toll interacting protein (TOLLIP)	Toll interacting protein TOLLIP interacts with components of the Toll-like receptors (TLR), regulating innate immunity. In IPF patients three SNPs play an important role: two of them may be implicated in pathogenesis (rs111521887, rs5743894), whereas the last seems to be protective (rs5743890).
	T-cells		T-cells are the prevalent immune population in IPF lung biopsies, particularly close to fibroblastic foci. Interleukin-13 (IL-13) produced by T-cells is a regulator of ECM deposition, and may have a pro-fibrotic effect if over-expressed.

## Treatment

Since the conduction of the first trial almost 30 years ago [76], the landscape of pharmacological treatment of IPF underwent major changes, reflecting the advances in the understanding of its pathogenetic mechanisms, the standardization of the diagnostic criteria and the improvements in the design of larger randomized clinical trials (RCT). The body of evidence against the safety and efficacy of drugs targeting the inflammatory and immune responses, or interfering with the coagulative asset [77–79] ultimately led to a strong recommendation against their use in the last update of the clinical practice guidelines on the treatment of IPF [52]. On the other hand, the first successful late phase RCT [49, 80] led to the worldwide approval of two agents capable of modifying the natural course of IPF - pirfenidone and nintedanib – revolutionizing the medical management of IPF [81].

## Pirfenidone and Nintedanib

Pirfenidone, an orally administered pyridine, demonstrated combined anti-inflammatory, anti-oxidant and anti-fibrotic actions both in vitro and in animal models of pulmonary fibrosis, consisting in the regulation of the expression of TGF-β and inhibition of fibroblast and collagen synthesis. However, the precise mechanism of action remains unknown.

Four placebo-controlled randomized trials [82–84] explored and confirmed the beneficial effect of pirfenidone in IPF patients. The results of a pre-specified pooled data analysis incorporating data from the phase 3 trials supported the efficacy of pirfenidone towards the reduction of overall and IPF-related mortality, although rates of death did not differ significantly in the individual prospective trials.Overall, the use of pirfenidone in the reported studies was associated with adverse events of generally mild to moderate intensity, such as gastrointestinal symptoms (nausea, dyspepsia), raised liver function tests and photosensitivity. The favourable safety profile and good tolerability of pirfenidone have been confirmed by post-authorization data provided by recent interim reports from internationalopen-label extension studies [85, 86]. Findings from several single-center European and Japanese studies have alsocontributed to confirmlong-term tolerability and also efficacy, sometime showing a trend toward stabilization of the disease in a significant proportion of treated patients [87–90].

Nintedanib is a multiple inhibitor of tyrosine kinase receptors implicated in lung fibrosis pathogenesis, including PDGF receptors α and β, VEGF receptors 1, 2 and 3, and FGF receptors 1, 2 and 3 [91], which was shown to prevent the development of lung fibrosis in the bleomycin murine model [92]. Nintedanib at a dose of 150 mg given twice daily showed efficacy in reducing in the rate of functional loss in phase 2 an 3 trials [93, 49],prompting the approval of the drug for use in patients with mild-to-moderate IPF.Gastrointestinal side effects (diarrhoea, nausea, anwere the most common side effects in the treated groups.

Evidence from real-life experiences of nintedanib use is very limited. Data from a German multicenterstudy on the compassionate use program of nintedanib in IPF [94] reported that after 6 months from the start of treatment with nintedanib most patients reached clinical and functional stability, including a subgroup ofpatients who had progressed under previous treatmentwithpirfenidone.

### Therapeutic approach of IPF: A practical guide

For the first time after decades of disappointing results, physicians have now two equally effective treatment options to offer to patients with IPF. Translating the results of the trials into clinical practice proved to be not straightforward though, and some uncertainties are still limiting the management of the heterogeneous IPF population.

The timing for starting treatment might represent the first challenge for physicians approaching those patients newly diagnosed with IPFwho are asymptomatic and have little or no functional impairment. In fact, it is unclear whether they might benefit from any treatment since there is no marker to ascertain the future clinical course of these patients. Recent post-hoc analyses of pooled data from the late phase trials on the efficacy of both nintedanib and pirfenidone showed that in the placebo arms subgroups of patients with more preserved lung function at baseline had a similar rate of progression of the disease as compared to patients with more impaired lung function, while the treated subgroups showed to receive the same benefit from both drugs [95, 96]. This evidence would suggest that an early commencement of anti-fibrotic therapy in patients with IPF is advisable regardless of their functional impairment at baseline. Indeed, until effective markers for predicting disease course are validated, risks and potential benefits of an early start of treatment should be evaluated in the single individual, also keeping an eye on the high costs of the available therapies.

Duration of treatment represents a matter of debate, as it is unknown whether pirfenidone or nintedanib maintain their efficacy for periods longer than 2 years and whether they should be discontinued in those patients experiencing significant disease progression, as there are no markers of response to treatment available yet. A subgroup analysis of data from the CAPACITY and the ASCEND trials showed that in patients who progressed significantly during treatment (predicted FVC decline > 10% after 6 months), those who continued pirfenidone had a lower risk of subsequent FVC decline or death [97]. As for Nintedanib, first evidence on the efficacy of long-term treatment has been recently provided by the open-label INPULSIS-ON trial, evaluating the safety and efficacy of nintedanib 150 mg twice daily in patients who completed an INPULSIS trial. Such analysis suggested that patients continuing or starting nintedanib in INPULSIS-ON declined similarly to patients treated with nintedanib in INPULSIS, suggesting that the efficacy of nintedanib is kept for up to 3 years [98].

This trial also allowed to enter patients with more severe disease (i.e., FVC ≤ 50% predicted, and exclusion criteria for most randomized trials in IPF) and will hopefully clarify whether the beneficial effects of these drugs may be generalized to the whole IPF population. The interim analysis showed that patients with FVC ≤ 50% and > 50% predicted at baseline had a similar decline in FVC to week 48, suggesting that nintedanib may offer similar benefits in patients with advanced disease [98]. However, only 24 patients with FVC ≤ 50% predicted were included in the analysis, as such these finding are not conclusive. Nevertheless, with the sole exception of the America Food and Drug Administration Agency, regulatory health agencies excluded this population from the indication to treat with pirfenidone and nintedanib, and stronger post-marketing surveillance evidence is needed to change the current regulations.

With two drugs available, which should be chosen when starting treatment? Indeed, pirfenidone and nintedanib appear to have comparable efficacy and tolerability, as well as a partially overlapping spectrum of potential side effects [49, 99]. As such, the initial choice should be based on the careful consideration of the patient’s features, including comorbidities, concomitant medications, and personal preferences. The replacement of the first agent should be considered when side effects are not tolerable, while it does not seem convenient to interrupt either anti-fibrotic treatment when disease progression is evident, as both pirfenidone and nintedanib seem to maintain efficacy over several years. Very few data are available on patients experiencing such therapy shift. Retrospectively, a small population of patients switching to nintedanib from pirfenidone treatment has shown that nintedanib may be better tolerated, but no conclusions can be drawn from this limited evidence [100].

When available and appropriate, the option of participating in a clinical trial should always be considered and discussed with the patient. This can give access to new, potentially beneficial therapies and gives the patients the opportunity to play an active role in their management and to be followed by expert medical staffs in specialized centers.

Following the example of most fields of respiratory medicine, such as asthma, chronic obstructive pulmonary disease, pulmonary hypertension and lung cancer, combination therapy that includes the use of different molecules in a synergic has a strong rationale [101]. Indeed, the association of drugs with proven efficacy or, alternatively, the addition of a promising agent to a background effective therapy are likely to represent the future of pharmacological therapy in IPF. To date however not much is known about the interactions between the two approved drugs when administered together, both in terms of tolerability and efficacy. The combination seemed safe in a small Japanese study in a small cohort of 50 patients [102], which suggested that exposure to nintedanib decreased when added to pirfenidone, while the latter was not affected. More recently, thesafety and tolerability of a combination regimenhas been supported by open-label, 12 weeks-randomized trial of nintedanib with add-on pirfenidone, compared with nintedanib alone. Interestingly, no pharmacokinetic interaction between nintedanib and pirfenidone was observed [103]. Favourable safety profiles have also been observed in an interimanalysis from a 24-week single-arm study on the safety and tolerability of pirfenidone with add-on nintedanib after at least 12 weeks of combined treatment [104].

A larger, multicenter phase 2 open-label, multiple dosing trial to investigate the pharmacokinetics of nintedanib and pirfenidone when administered separately or in combination has been recently conducted in the UK (https://clinicaltrials.gov/ct2/show/NCT02606877 Identifier: NCT02606877). For the time being, it is advisable to avoid the concomitant use of the two drugs given the risk associated with the partly overlapping side effect profiles.

### Supportive care

Neither pirfenidone or nintedanib succeeded to demonstrate a survival benefit in IPF, nor they proved to improve the symptoms of these patients, often burdened by a heavily impaired quality of life and repercussions on psychological and emotional levels [105, 106]. Whilst there is no definite evidence regarding the best timing for initiation of supportive care in IPF [107], the unpredictability of the disease would suggest that palliative care should be integrated early and regarded as a standard of care to provide relief from the symptoms and anxiety related to fear of these symptoms [108]. Indeed, the level of provision of supportive care seems to be not adequate in IPF. A retrospective investigation of decedents patients with IPF showed that only a minority of patients who died in a hospital actually received palliative care before admission [109]. Recently,a few well-designed qualitative studies found significant gaps between the perceived needs of patients and their carers and the quality and timing of information provided by physicians about the meaning of the disease-centered assessments, disease prognosis and its management. Such evidence highlights the requirement of a pragmatic, continued needs assessment and the identification of triggers to refer patients to supportive and palliative care [110, 111]. A supportive approach to these patients might be best delivered through the joint efforts of a well-coordinated multidisciplinary team including doctors, nurses and social workers, with the main goal of improving the quality of life of these patients [112].

Chronic cough affects up to 80% of patients and has a significant impact on quality of life [113]. Physicians should identify possible triggering factors or comorbidities, such as gastroesophageal reflux disease (GERD), obstructive sleep apnoea (OSA), infections or ACE inhibitor use. This symptom is often refractory to conventional anti-tussive treatments [114]. Oral corticosteroids and opiates are often used in clinical practice, but the benefit is unclear [115]. A single center double-blinded study of thalidomide showed an improvement in quality of life, but the significant side effects reported - such as dizziness and neuropathy - seem to exclude the routine use of this agent for treating cough in IPF [116]. A subgroup analysis of a phase 3 trial of pirfenidone in Japan showed the potential efficacy of the anti-fibrotic drug in reducing cough, and the evaluation of this symptom through validated measurements could be an endpoint in future trials [117].

Functional de-conditioning is also very common in patients with IPF. As such, respiratory rehabilitation has been proposed as a valid intervention in these patients and demonstrated to improve 6-min walk distance and shortness of breath [118], and seems to reduce anxiety and depression and enhance the quality of life, other than to maintain musculoskeletal conditioning [119]. Nevertheless, the beneficial effect does not seem to persist after 6 months [120]. A study showed that subjects did not keep on with exercise at home following a rehabilitation program of 3 months, which highlights the importance of compliance in rehabilitation [121].

Patients with IPF may present with hypoxemia during exercise, sleep or even at rest as a result of impaired gas exchange due to disease progression with or without concomitant conditions such as pulmonary hypertension. Despite there is no definite evidence of its beneficial effects in IPF [122], supplemental long-term oxygen therapy is required to contrast the detrimental effects of low oxygen levels, that may impact on symptoms, the performance of daily activities and therefore overall quality of life [123].

The need of psychological support in IPF patients is considered to be comparable to that of cancer patients, with depression related to shortness of breath, fatigue, and cough being reported in up to 25% of IPF patients. Nevertheless, the impact of interventions aimed to reduce emotional disturbance such as psychological counselling, support groups or mindfulness programs has never been measured in clinical studies, and the provision of such services in clinical practice is poor [123, 124]. In patients with advanced lung disease, psycho-educational intervention and cognitive behavioural therapy seem to help develop coping strategies and feel less isolated, as described in a pilot study of a psycho-educational intervention program (PRISIM) [106]. Participation to support groups might help patients and carers reduce the psychological burden of the disease through sharing feelings and experiences, and represent a compelling opportunity for providing practical information about IPF and its management. Support networks have been recently developed in IPF thanks to the foundation of regional networks of specialist centers [124].

Another area of interest in supportive care is represented by mindfulness techniques, originally developed by J Kabat – Zinn in 1979 to integrate meditation with clinical and psychological practice. Mindfulness is a term indicating a state of mental presence and attention to the present moment, which can help discriminate positive thoughts and emotions from the negative ones often leading to repercussions on the emotional level. In patients with breast cancer, mindfulness-based programs have demonstrated to be effective in reducing anxiety and depression [125]. In asthmatic patients, this programs showed to improve quality of life by promoting coping strategies and reducing reactivity to dyspnoea, irrespectively to impairment of lung function [126]. Recently, a single-center pilot study suggested that Mindfulness-based programs are feasible in patients with ILD and might have a positive effect on mood [127].

In conclusion, non-pharmacological interventions and supportive care might help reduce the burden of illness in IPF patients and their carers and should be promoted since the earlier stages of the disease. A better knowledge of the impact of such strategies and their standardization is warranted though to deliver an appropriate, individualized approach.

### Treatment of comorbidities

Multi-morbidity is frequent among IPF patients, who have a median age of 66 years at diagnosis and present risk factors shared with several health conditions. Most common respiratory comorbidities include chronic obstructive pulmonary disease (COPD), lung cancer, pulmonary hypertension, obstructive sleep apnoea, while non-respiratory comorbidities include ischemic heart disease and gastro-oesophageal reflux [128] The greatest challenge for clinicians is probably to understand to which extent these comorbidities might impact the clinical course of the disease and affect prognosis. Unfortunately, little is known about the real prevalence and burden of comorbidities in patients with IPF although a recent systematic review tried to clarify the prevalence and prognostic implications of various comorbidities in IPF patients across 126 studies [129]. Data are also limited as to the correct management of comorbidities in IPF, and more robust evidence from prospective multicenter studies are required to determine the impact of conventional treatment of comorbidities in IPF population and to evaluate effects of new anti-fibrotic medications [130].

The prevalence of COPD in IPF ranges from 6% to 67% across 23 different studies [129]. A syndrome called combined pulmonary fibrosis and emphysema (CPFE) has been recently described in patients with fibrosis in lower pulmonary lobes and coexisting emphysema in the upper regions [131]. Although pulmonary fibrosis seems to be the major determinant of the clinical course in these patients [132], CPFE has been proposed as a distinct disease entity for having different features as compared to both IPF and emphysema. Pulmonary function tests are usually characterized by relatively preserved lung volumes, due to the compensating effect of hyperinflation of emphysema on the reduced compliance produced by fibrosis, while the two conditions contribute together in producing a severe impairment of diffusing capacity of the lung for carbon monoxide(DLco). Most importantly, the prevalence of pulmonary arterial hypertension is higher in CPFE than in IPF alone (47–90% versus 31–46%) [133], with significant impact on mortality [134]. Should airflow limitation coexist in these patients, use of bronchodilators should be considered, although there is no definite evidence on their efficacy.

Lung cancer is more frequent in patients with IPF than in the general population, suggesting a predisposition to developing neoplasm in IPF. This could be explained both by common risk factors such as tobacco-smoking and by the sharing of pathogenic pathways and molecular alterations [135]. The prevalence varies from 3% to 48% across different studies, and it significantly affects prognosis, shortening survival by 2 years [136]. Remarkably, most patients with IPF and lung cancer are excluded from surgical options due to limited lung functionality and reduced exercise tolerance. Moreover, surgical procedures would also increase the risk of acute exacerbations of IPF, known to have a short-term mortality of approximately 50% [137].

Pulmonary hypertension (PH), usually defined as mean pulmonary arterial pressure (mPAP) ≥ 25 mmHg, is one of the conditions more frequently associated to IPF and has been widely demonstrated to increase mortality in this population [138, 139]. The absence of direct correlation between severity of PH and the extent of the underlying fibrotic disease implies that mechanisms other than hypoxia contribute to pulmonary vascular disease in IPF [140]. Despite the clear prognostic implications, the benefits of treating this condition in IPF patients remain unknown. Overall, studies investigating PH-directed therapies failed so far to prove efficacy in IPF [141–144], although Sildenafil, a phosphodiesterase-5 inhibitor, showed some positive effects on DLco, quality of life and symptoms in patients with advanced IPF [145, 146]. Indeed, the negative results obtained so far might also be due to intrinsic limitations of trial design rather than to a real lack of efficacy of the drugs being tested [147], and further evidence is needed to clarify the potential benefit of these treatments in a more targeted population of patients where PH is the primary driver of poor outcome.

Moderate to severe OSA affects up to 65% IPF patients [148, 149]. Patients with IPF, OSA and sleep-related hypoxemia had a worse prognosis and disease progression rates than patients with IPF alone [150]. Small, nonrandomized studies demonstrated improved quality of life in patients with IPF, and concomitant OSA treated with continuous positive airway pressure (CPAP) [151, 152].

GERD is common in IPF patients, and the use of proton pump inhibitors (PPI) demonstrated to improve survival in retrospective studies [153, 154]. Based on such evidence and the low cost of therapy and risk of side-effects, in the most recent update of the guidelines for the treatment of IPF [52], anti-acid treatment was recommended in most IPF patients.

The coexistenceof coronary artery disease deservesattentionis observed in up to 30% of patients with IPF and increases mortality [155]. The management of coronary artery is challenging since complications of invasive therapy are more frequent in IPF patients due to their performance and respiratory status [156]. Consequently, the majority of these patients are being treated with standard medical therapies. However, patients with IPF should be evaluated for coronary artery disease from the clinical and radiological point of view. Coronary artery calcification at chest CT scan represents a useful tool and a potential screening tool to detect at-risk patients [157].

Anxiety-depressive disorder considerably affects patients with IPF with a prevalence of 11–50% [158]. Depression has a harmful impact on quality of life and reduces adherence to treatment of these patients [159]. For this reason, patients with a new diagnosis of IPF should be screened for depression and anxiety and merit an early referral to a psychiatric consultation [160].

## Conclusions

Nowadays, substantial advances have been achieved in the understanding of IPF pathogenesis and in the therapeutic possibilities that can be offered to patients. Nevertheless, major issues regarding diagnosis and management are still open. The process leading to a confident diagnosis of IPF is far from being straightforward. Currently, only morphological data coming from HRCT or SLB are available for discussion, and their interpretation can significantly vary between clinicians, even if experts. Several different genetic and biological markers have been proposed for aiding diagnosis and prognosis [161], however their clinical utility has remained elusive so far. It is a common belief that in the next future blood or lung specific molecular biomarkers, reflecting disease activity and behaviour, will be incorporated in the diagnostic process [67]. Nevertheless, at the moment it is hard to foresee their weight in the diagnostic workup, especially in comparison to morphological features. The management of IPF patients, after the approval of two new effective therapies, has dramatically changed over the last years. Both pirfenidone and nintedanib showed outstanding efficacy in reducing the functional decline in IPF, although they do not seem capable of improving the survival of these patients in a significant way. The discovery of effective pharmacotherapies strongly encouraged the research for new drugs, and many different molecules are currently investigated in the context of phase I and phase II clinical trials [162]. Further researches are also focused on combination trials with the existing antifibrotic agents [163] and their use in progressive fibrosing pneumonias other than IPF. Nevertheless, patients with IPF daily struggle against a variety of symptoms like chronic cough and shortness of breath, and are frequently affected by several comorbidities that should be systematically identified and addressed. As such, these patients should be comprehensively managed by adding non-pharmacological interventions with the goal of improving health-related quality of life, and not only lung function decline over time [164].
